# Improving productivity of Sesbania pea in saline soils by enhancing antioxidant capacity with optimum application of nitrogen and phosphate combination

**DOI:** 10.3389/fpls.2022.1027227

**Published:** 2022-11-02

**Authors:** Guanglong Zhu, Yunming Xu, Zhenran Xu, Irshad Ahmad, Nimir Eltyb Ahmed Nimir, Guisheng Zhou

**Affiliations:** ^1^ Joint International Research Laboratory of Agriculture and Agri-Product Safety, The Ministry of Education of China, Yangzhou University, Yangzhou, Jiangsu, China; ^2^ Jiangsu Key Laboratory of Crop Genetics and Physiology/Jiangsu Key Laboratory of Crop Cultivation and Physiology, Agricultural College of Yangzhou University, Yangzhou, China; ^3^ Jiangsu Co-Innovation Center for Modern Production Technology of Grain Crops, Yangzhou University, Yangzhou, China; ^4^ Faculty of Agriculture, University of Khartoum, Khartoum, Sudan

**Keywords:** saline soil (solonchak), sesbania pea, growth traits, biomass yield, antioxidant capacity

## Abstract

Salinity stress is one of the major constraints to plant growth and crop production. Optimum fertilizer management is essential for promoting crop growth and productivity in saline soils. A field experiment was conducted to study the effects of nitrogen and phosphate combination on sesbania pea (*Sesbania cannabina* (Retz.) Poir.) growth and associated physiology in saline soils. Three N rates (N1: 240 kg·ha^-1^, N2: 360 kg·ha^-1^, N3: 480 kg·ha^-1^) and two P rates (P1: 120 kg·ha^-1^, P2:180 kg·ha^-1^) were arranged in this study using a RCBD with 3 replicates. The application of N and P fertilizers significantly improved plant growth and associated physiological traits of sesbania pea. Plant height (*P*=0.0001), fresh biomass weight (*P*=0.0006), dry biomass weight (*P*=0.0006), relative growth rate (RGR) (*P*=0.005), chlorophyll (*P*=0.002), peroxidase (POD) (*P*=0.0003), catalase (CAT) (*P*=0.0001), superoxide dismutase (SOD) (*P*=0.0001) and soluble protein (*P*=0.0053) were significantly increased, and the maximum values were consistently produced under N2P2 combination at each growth stage. On the contrary, malondialdehyde (MDA) was prominently decreased by N and P fertilizer application (*P*=0.0029), and the lowest values were all produced under N2P2 combination. The highest values of plant height, fresh biomass weight and dry biomass weight were recorded on the 163rd day after seeding (DAS). The highest RGR and MDA content were determined on the 141st DAS. The highest chlorophyll content, CAT and SOD activity, and soluble protein content were recorded on the 110th DAS, and the highest POD activity was at 47 DAS. This study suggested that the optimum N and P fertilizer combination was N2P2 (360 kg·hm^-2^ N + 180 kg·hm^-2^ P), which was superior in promoting growth and biomass yield with enhanced antioxidant capacity of sesbania pea in saline soils.

## Introduction

Cultivated area is shrinking more quickly than ever due to rapid economic and social development and urbanization. Under this circumstance, much importance is attached to the development and utilization of saline sols. Globally, approximately 1 billion ha of lands are now affected by salinity(Hopmans et al., 2021). In China alone, about 100 million ha of lands are salinized, over half of which can be used to develop crop production(Zhu et al., 2020). The improvement and utilization of saline lands for crop production has become a national strategy to ensure food security in China (Zhen and Wang, 2015).

However, salt stress is one of the major constraints to plant growth and crop production (Islam et al., 2013). Saline soils have high levels of soluble salts and exchangeable sodium (Na^+^) and chlorine (Cl^−^) which usually pose stress and damage to plants (Mumtaz et al., 2018). Salt stress includes osmotic stress, ionic imbalances, and secondary stresses like nutritional imbalances and oxidative stress for glycophytes (Deinlein et al., 2014). In these stress processes, Na^+^ is the dominant ion causing toxicity (Farooq et al., 2015). In saline soils, glycophyte crops, such as wheat (*Triticum aestivum* L.), maize (*Zea mays*) and rice (*Oryza sativa* L.) cannot grow well or achieve reasonable productivity due to salt stress. For example, the yield reduction could be reached to 50.0%, 37.8% and 31.3% under 0.3%-0.5% salinity stress for wheat (Rahaman et al., 2012), maize (Zheng et al., 2010) and rice (Zhu and Fan, 2021), respectively. Therefore, the improvement of saline soils is the first step for farming in these soils.

At present, four major approaches are used to improve saline soils, including hydrotechnical amelioration, physical amelioration, chemical amelioration, and biological amelioration. Through hydrotechnical amelioration, the soluble salts in the saline soils can be infiltrated along the capillary tubes to reduce salt content in surface soil by artificial irrigation and washing (Zhang et al., 2016). Through physical amelioration, saline soils can be levelled, loosened, covered, or replaced with foreign non-saline soils (Zhang et al., 2016). Some chemicals, such as ardealite, can be used to reduce the content of salts in saline soils. But this method may bring foreign pollutants into the saline soils. Through biological amelioration, proper halophytes or salt-tolerant crops are planted to ameliorate saline soils (Guo et al., 2019a). Among the above-mentioned methods, biological amelioration is most widely used because it has the advantages of economy, efficiency, and sustainability (Szabolcs, 1995; Wang et al., 2011). However, some salt-tolerant crops still face the difficulties in germination, seedling establishment, and yield formation when they are planted in saline soils for biological amelioration (Szabolcs, 1995). In this sense, the screening and breeding of salt-tolerant crops varieties and the development of salt-tolerant cultivation techniques are the per-conditions for biological amelioration (Pinthus, 1974; Wang et al., 2011).

Sesbania pea (*Sesbania cannabina* (Retz.) Poir.) is a rapid-growing annual herbal shrub that is widely adaptable to adverse environment conditions such as high salinity (Guo et al., 2019a). It grows extensively in Asia, Australia and Africa as an important forage crop and green manure crop. As an excellent feed crop, it contains high content of vitamins, fat, carbohydrates and various minerals. It is also remarkably effective in improving soil fertility and reducing soil salt content (Guo et al., 2019b). It has been widely reported as a pioneer crop for improving saline-alkali lands (Guo et al., 2019b). The growth and yield of sesbania pea could be enhanced significantly in saline soils by genetic improvement for salinity tolerance and cultivation techniques, such as appropriate nitrogen application and planting density (Guo et al., 2019b).

Of all the techniques to manage salinity, fertilizer management is one of the most effective strategies (Ladha et al., 2005). The salt tolerance of crops can be significantly improved by rational application fertilizer. Almost all surveys showed that seed yield of oat (*Avena sativa* L.) was increased substantially by appropriate nitrogen fertilizer (200 kg ha^-1^) under salt stress, which was mainly due to it enhanced photosynthesis and nitrogen accumulation (Song et al., 2019). Besides nitrogen fertilizer, phosphorus and potassic fertilizer can alleviate salt stress to crop in saline soils (Manimaran and Poonkodi, 2014). In addition, the promoting effects of N combined with P was better than N or P solely application, which was attributed to it maintained a higher leaf area, chlorophyll content and net photosynthetic rate (Xin et al., 2010). As for sesbania pea, limited research was focus on salt-tolerant cultivation techniques. It was reported that plant height, biomass, pod number per plant, seed number per pod, seed yield and N uptake were remarkably improved with increasing N rate from 180 to 360 kg ha^-1^ (Guo et al., 2019b). Although different types of fertilizers have been proved to enhance plant growth and relieve salt stress separately, the mixture effects of the fertilizer, especially the effect of nitrogen combined with phosphorus on growth, yield and associated physiological mechanism of sesbania pea is still not fully understood.

We hypothesized that appropriate combination of N and P fertilizers can effectively improve the growth and yield of sesbania pea through bettering physiological mechanism. Therefore, a field study was conducted with three N rates and two P application rate to: (1) evaluate the effects of N combined with P fertilizers on sesbania pea productions in the saline soils; (2) explore the associated physiological mechanism of N combined with P fertilizers effects on sesbania pea growth; and (3) select the applicable rate of N and P fertilizer for promoting sesbania pea production in saline soils.

## Materials and methods

### Plant materials and experimental arrangement

**Figure 1:**
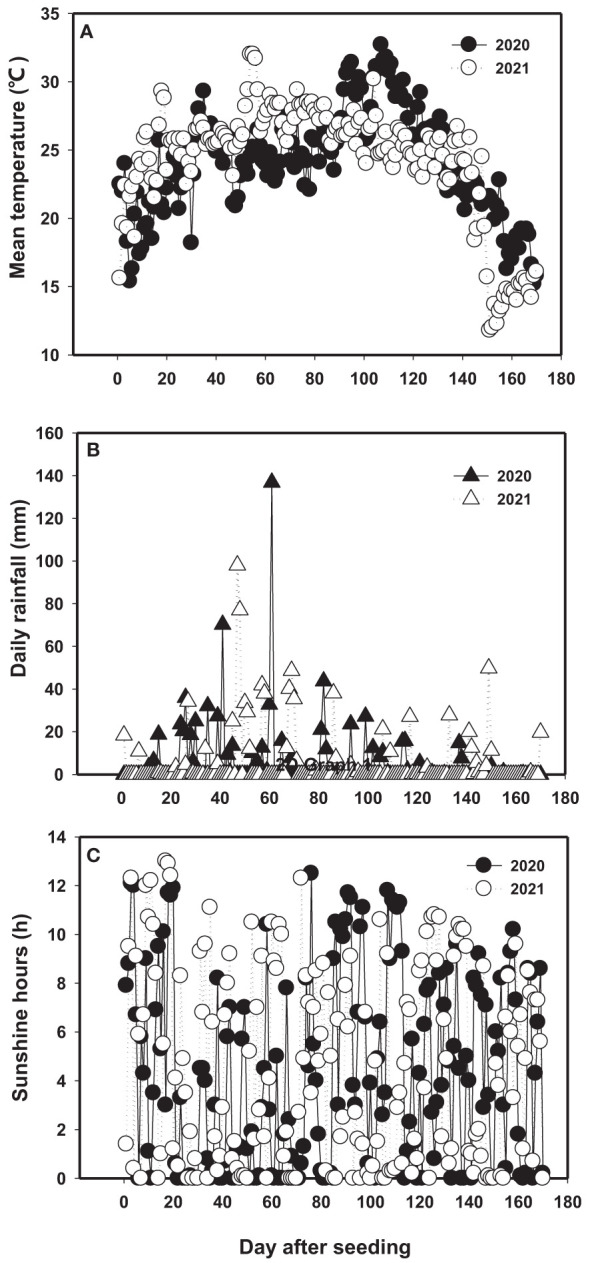
Daily mean temperature **(A)**, daily rainfall **(B)** and daily sunshine hours **(C)** in 2020 and 2021 during growth periods (from May to October) at experiment site of Dafeng district.

A field study was conducted on Coastal Forest Farm of Dafeng, Dafeng county (33°20′N, 120°47′E), Yancheng City, Jiangsu Province, China in the two growing seasons of 2020 and 2021. The soil in the experimental field had the texture of clay loam, and the chemical composition as showed in **Table 1**. It belongs to slight-middle level saline soil. The average daily temperature during the growing season (from May to November) was 21.9°C in 2020 and 22.0°C in 2021 (**Figure 1A**). The daily rainfall was 4.20 mm and 4.54 mm in 2020 and 2021, respectively (**Figure 1B**). The mean daily sunshine hour from May to October was 4.50 and 4.93 h day^−1^ in 2020 and 2021, respectively (**Figure 1C**). The average daily temperature was 2.64°C higher in 2020 than in 2021 during 100-120 day after seeding (DAT), but 2.33°C lower during 50-90 DAT. The daily mean rainfall and sunshine hour were 8.1% and 9.6% higher in 2021 than in 2020, respectively (**Figure 1**).

**Table 1:** Chemical composition of soil in saline soils in this study, including Total N (Kjeldahl), Available P (Mehlich), Available K (flame spectrophotometry), Organic matter (Potassium dichromate oxidation volumetric method), pH (electrode method), and EC (1:5).

Parameters	Chemical composition of soil						
	Total N (g kg^–1^)	Available P (mg kg^–1^)		Available K (mg kg^–1^)	Organic matter (g kg^–1^)	pH	EC (mS cm^-1^)
Value	0.73	1.42		279	19.75	8.6	10.87

EC, electrical conductivity. The composition of soil was assayed by the institute of soil science and nutrition, Jiangsu Academy of Agricultural Sciences.

The seeds of sesbania pea were provided by the Company of Coastal Forest Farm of Dafeng. Healthy and uniform size seeds were sown by broadcast at the seeding rate of 45 kg hm^-2^ on May 15, both in 2020 and 2021. The three N rates and two P fertilizer rate were arranged as N1: 240 kg·ha^-1^, N2: 360 kg·ha^-1^, N3: 480 kg·ha^-1^, P1: 120 kg·ha^-1^, P2:180 kg·ha^-1^. Urea containing 46% N was used as the nitrogen source, and the form of the P fertilizer was calcium superphosphate (containing 12% P_2_O_5_) was applied as phosphate source. All the amount of fertilizers was equally separated two parts and applied as basal fertilizer before sowing and topdressing fertilizer at seedling stage (47 DAS, day after seeding), respectively. The field study was arranged in randomized complete block design with 3 replicates. The plot size was 30 m^2^ (2 m × 15 m). Other field practices were used in conformity with local recommendations to avoid yield losses.

### Observations and measurements

Growth and antioxidant physiological parameters were assessed on the 47th DAS (seedling stage), 72nd DAS (squaring stage), 110th DAS (blooming stage), 141st DAS (pod bearing stage), and 163rd DAS (maturity stage). Growth parameters including plant height, relative growth rate (RGR), and biomass yield (fresh weight and dry weight) were determined. Antioxidant physiological parameters determined were the activities of peroxidase (POD), catalase (CAT), superoxide dismutase (SOD), and the contents of chlorophyll, soluble protein and malonaldehyde (MDA).

### Agronomic parameters

About 5 m^2^ of sesbania pea plants were harvested from each plot to measure fresh biomass yield (fresh weight) and dry biomass yield (dry weight) on the 47th, 72nd, 110th, 141st, and 163rd DAS. After measurements of plant height and fresh weight, the sampled plants were oven-dried at 80°C to constant weight for dry biomass determination. After that, the relative growth rate (RGR) was calculated using the following formula(Zhu et al., 2020)


$${\text{RGR(kg hm}}^{-2}{\text{d}}^{-1}\text{) = (W2-W1)/(T1-T2)}$$

where W1 and W2 were the dry biomass measured at T1 and T2 stage, respectively. As for the RGR from 0-47 DAS, the T1 was started from the seeding time, so both the W1 and T1 was recorded as 0.

### Physiological parameters

At each growth stage, the upper expanded leaves from 5 sesbania pea plants in each plot were sampled and immersed in liquid nitrogen immediately, after that stored in an ultra-low temperature refrigerator (-80 °C) for physiological assays.

Chlorophyll content: About 0.5 g leaf samples were extraction in 80% (v/v) aqueous acetone overnight. The optical density was measured at 663, 652, and 645 nm using a spectrophotometer (Beckman Coulter, Inc. CA, USA) (Guo et al., 2019b). The contents of chlorophyll a and chlorophyll b were calculated by the following equations:


$$\text{Chl }a\;(mg\;g^{-1}\text{FW) = (12}.21\times Abs663-2.81\times Abs646)\times \text{V/(1000}\times \text{W)Chl }b{\text{(mg g}}^{-1}\text{FW) = (20}.13\times Abs646-5.03\times Abs663)\times \text{V/(1000}\times \text{W)}$$

where Abs is the absorbance, V is the final volume of the extract (mL) and W is the weight of the leaf sample (g) used.

POD activity: About 0.1 g fresh leaf was ground in 3 ml of 0.1 mol L^-1^ phosphate buffer (pH 7.0), the extraction was centrifuged at 18 000 × g at 4°C for 15 min. The supernatant was used to assay the enzyme of POD at 430 nm after oxidized with o-diphenylamine Phosphate buffer (0.1 mol L^-1^, pH 6.5) was placed in colorimetric dishes containing enzyme extract. Then, 0.2 ml 0.2 mol L^-1^ H_2_O_2_ was added and mixed, and the absorbance per minute was recorded. The POD activity unit expressed as the rate of increase in absorbance per minute per milligram of protein (Assaha et al., 2017).

CAT activity: 0.1 g of fresh leaf was homogenized in 5 ml assay mixtures containing 2.9 ml substrate solution (30% hydrogen peroxide in 50 mmol L^-1^ potassium phosphate buffer) and 0.1 ml of enzyme extraction. The decomposition of H_2_O_2_ was stopped by adding 2 ml potassium-dichromate (5%) to the mixed solution. The absorbance reading was measured at 620 nm. Enzyme-specific activity is expressed as mmol L^-1^ H_2_O_2_ (mg protein) oxidized per minute (Zhu et al., 2022).

SOD activity: About 0.2 g fresh leaf was milled and homogenized in 5 ml 100 mmol L^-1^ potassium phosphate buffer (pH 7.8), which containing 0.1 mmol L^-1^ EDTA, 0.1% Triton X-100 and 2% polyvinyl pyrrolidone. The extraction was centrifuged with 15000 × g at 4°C for 15 minutes, the supernatant was used for determination. The total volume of 3 ml of the assay mixture which contained 50 mmol L^-1^ sodium carbonate/sodium bicarbonate buffer (pH 9.8), 0.1 mmol L^-1^ EDTA, 0.6 mmol L^-1^ epinephrine, and enzyme were measured at 560 nm. One unit of SOD activity is expressed as the amount of enzyme required to cause 50% inhibition of epinephrine oxidation (Jini et al., 2017).

Soluble protein content: About 0.5 g fresh leaf sample was frozen and ground to a fine powder with liquid nitrogen and extracted with 5 ml of ice-cold potassium phosphate buffer with 1 mM ascorbic acid (pH 7.8). The extract was centrifuged at 20,000 × g and 4°C for 20 min. The supernatant layer was used for protein assays (Kawano and Kypta, 2003).

MDA content: About 0.5 g of fresh leaf was ground in 0.1% trichloroacetic acid (TCA), then mixed and centrifuged at 12,000 × g for 15 minutes. After that, 1 ml supernatant with 4 ml 0.5% thiobarbituric acid (containing 20% trichloroacetic acid) was heated at 95°C for 15 mins and then centrifuged at 10,000 × g for 15 mins. Then the sample was recorded the absorption at 600, 532 and 450 nm and MDA content were calculated (Seckin et al., 2009).

### Statistical analysis

Analysis of variance (ANOVA) was carried with Statistix 9.0 (Analytical Software, Tallahassee, FL), the mean values were compared based on the LSD test at *P*< 0.05 (LSD0.05) and the error bar represented standard error (SD). Statistix 9.0 (Analytical Software, Tallahassee, FL) was used to calculate the Pearson correlation coefficient and Sigmaplot 10.0 (SPSS, PointRichmond, CA) perform graphing.

## Results

The experimental field was coastal beach reclamation soil, which had the texture of clay loam with 0.73 g kg^–1^ total N, 1.42 mg kg^–1^ available P, 279 mg kg^–1^ available K, 19.75 g kg^–1^ organic matter, 1.48 g kg^–1^ soluble salt. The pH of the soil was 8.6 and soil EC (electrical conductivity) was 10.87 mS cm^-1^. It belongs to slight-middle level saline soil. The nutrition was relatively deficiency in saline soil and its implicated crop will significant response to fertilizer treatments. All the parameters are shown as the mean values of the 2-year experiments because the tendency of each parameter was similar in each year.

### Plant height

**Figure 2:**
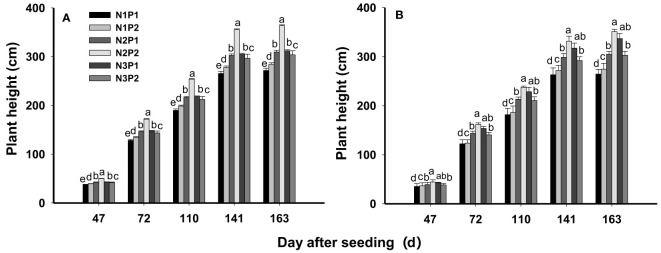
Effects of different nitrogen and phosphorus combinations on plant height of sesbania pea at different growth stages in saline soils. Different letters indicate significant differences between different treatments at same growth stage at P<0.05 level. **(A)**: 2020, **(B)**: 2021.

Plant height was significantly affected by N, P fertilizer treatments and their interactions at all growth stages (*P* = 0.0006). Plant height was prominently enhanced by N and P application. In general, plant height was significantly improved with increased N rate under P1 (*P*=0.0035), and the highest plant height was showed under N3 with mean value of 39.1, 134.9, 199.8, 284.0 and 288.2 cm at each growth stage. On the average, the plant height under N3P1 was 4.4% and 19.6% higher than N2P1 and N1P1 across the growth periods and years, respectively. However, under P2, plant height was increased with increased N rate and the maximum value was performed under N2 with 47.2, 166.7, 245.7, 343.3 and 357.2 cm. The plant height of N2P2 was higher than N3P2 and N1P2 by 17.1% and 27.0% at all growth stages, respectively. Overall, the highest plant height was showed in N2P2 (**Figure 2**).

### Fresh biomass yield

Fresh biomass yield was significantly affected by N, P treatments and the interaction between N and P fertilizers at each growth stage (*P* = 0.0006, **Table 2**). The N and P application significantly increased fresh biomass yield in saline soil. Under P1, fresh biomass yield was significantly improved with increased N rate (*P*=0.0004), and the highest fresh biomass yield was produced under N3 with 0.38, 7.06, 24.90, 40.73 and 42.97 Mg ha^-1^ at each growth stage. Compared with N1P1, the fresh biomass yield was increased by 25.9% and 48.3% under N2P1 and N3P1 on the 47th DAS, by 23.8% and 37.9% on the 72nd DAS, by 18.6% and 30.9% on the 110th DAS, by 19.9% and 34.8% on the 141st DAS, and by 22.5% and 36.7% on the 163rd DAS, respectively. Moreover, under P2, the fresh biomass yield was increased with increasing N rate and the maximum value was performed under N2. The fresh biomass yield of N2P2 and N3P2 was 44.8% and 10.3% higher than N1P2 on the 47th DAS, 39.1% and 10.1% on 72nd DAS, 33.8% and 9.4% on the 110th DAS, 36.8% and 8.2% on the 141st DAS, and 37.8% and 10.0% on the 163rd DAS, respectively. Overall, the maximum fresh biomass yield of sesbania pea was showed under N2P2 among all the treatments (**Table 2**).

**Table 2:** Effects of different nitrogen and phosphorus combinations on fresh biomass yield of sesbania pea at different growth stages in saline soils in 2020 and 2021.

Treatment	Fresh biomass yield(Mg·ha^-1^)									
	47 DAS		72 DAS		110 DAS		141 DAS		163 DAS	
	2020	2021	2020	2021	2020	2021	2020	2021	2020	2021
N1P1	0.28e	0.23e	5.30e	4.95e	19.48e	18.56c	30.96e	29.47e	32.50e	30.41e
N1P2	0.31d	0.27d	5.88d	5.63d	21.64d	20.91bc	34.38d	33.15d	36.08d	34.19d
N2P1	0.34c	0.30c	6.37c	6.30c	23.44c	21.68bc	37.25c	35.24cd	39.09c	37.89c
N2P2	0.43a	0.40a	8.05a	7.96a	29.63a	27.32a	47.08a	45.32a	49.41a	47.37a
N3P1	0.38b	0.37b	7.05b	7.07b	25.94b	23.85b	41.22b	40.24b	43.26b	42.67b
N3P2	0.34c	0.30c	6.36c	6.31c	23.39c	23.17b	37.16c	35.90c	39.01c	38.29c
N	***	***	***	***	***	**	***	***	***	***
P	***	***	***	***	***	**	***	**	***	***
N*P	***	***	***	***	***	*	***	***	***	***

DAS, day after seeding. Different letters in each column indicate significant differences between different treatments at same growth stage at P<0.05 level. *significant at P<0.05, **significant at P<0.01, ***significant at P<0.001.

### Dry biomass yield

Dry biomass yield was significantly affected by N, P treatments and the interaction between N and P fertilizers at all the growth stages (*P*=0.0006, **Table 3**). Similar to fresh biomass yield, dry biomass yield was significantly increased with increased N and P rates in saline soils (*P*=0.0005). At the low level of P1, dry biomass yield was significantly improved with increased N rate (*P*=0.0048), and the highest value was showed under N3 at each growth stage. Compared with N1P1, the dry biomass yield was increased by 25.0% and 37.5% under N2P1 and N3P1 on 47th DAS, 30.3% and 49.3% on 72nd DAS, 15.2% and 29.7% on 110th DAS, 20.4% and 32.3 on 141st DAS, and 22.9% and 33.8% on 163rd DAS, respectively. In addition, at the high level of P2, the dry biomass yield was increased with increased N rate and the maximum value was performed under N2. The dry biomass yield of N2P2 and N3P2 was 30.0% and 10.0% higher than N1P2 on 47th DAS, 44.1% and 12.4% on 72nd DAS, 30.3% and 7.9% on 110th DAS, 42.1% and 8.2% on 141st DAS, and 40.6% and 8.1% on 163rd DAS, respectively. On the whole, the maximum dry biomass yield of sesbania pea was generated under N2P2 among the treatments (**Table 3**).

**Table 3:** Effects of different nitrogen and phosphorus combinations on dry biomass yield of sesbania pea at different growth stages in saline soils in 2020 and 2021.

Treatment	Dry biomass yield(Mg·ha^-1^)									
	47 DAS		72 DAS		110 DAS		141 DAS		163 DAS	
	2020	2021	2020	2021	2020	2021	2020	2021	2020	2021
N1P1	0.04e	0.04e	0.87e	0.65e	5.01e	4.53e	9.46e	8.21d	10.78e	9.29e
N1P2	0.05d	0.05d	0.96d	0.81d	5.56d	5.26c	10.50d	9.24d	11.97d	10.43d
N2P1	0.05c	0.05c	1.04c	0.94c	6.02c	4.97d	11.38c	9.89c	12.97c	11.70bc
N2P2	0.07a	0.06a	1.32a	1.23a	7.61a	6.39a	14.38a	13.67a	16.39a	15.11a
N3P1	0.06b	0.05b	1.15b	1.12b	6.66b	5.71b	12.59b	10.78b	14.35b	12.50b
N3P2	0.06b	0.05c	1.04c	0.95c	6.01c	5.67c	11.35c	10.01c	12.94c	11.26cd
N	***	*	***	***	***	***	***	*	***	**
P	***	***	***	***	***	***	***	***	***	***
N*P	***	***	***	***	***	***	***	***	***	***

DAS, day after seeding. Different letters in each column indicate significant differences between different treatments at same growth stage at P<0.05 level. *significant at P<0.05, **significant at P<0.01, ***significant at P<0.001.

### Relative growth rate

Relative growth rate (RGR) was significantly affected by N, P treatments and their interactions at all the growth stages (*P*=0.0050, **Table 4**). RGR was significantly increased with N and P fertilizer application in saline soil (*P*=0.0005). At the low level of P1, RGR was significantly improved with N rate increased, and the highest value was showed under N3 at each growth stage. At the high level of P2, RGR was increased with N application rate aggrandized and the maximum value was performed under N2. Besides that, at the rates of N1 and N2, RGR was promoted with P application increased, and showed the tendency of N1P2>N1P1, N2P2>N2P1. However, opposite tendency was showed at the rate of N3, which was N3P1>N3P2 at each growth stage. Obviously, the highest RGR was recorded under N2P2 at all growth stage (**Table 4**).

**Table 4:** Effects of different nitrogen and phosphorus combinations on relative growth rate (RGR) of sesbania pea at different growth stages in saline soil in 2020 and 2021.

Treatment	Relative growth rate(RGR)(kg·ha ^-1^·d ^-1^)									
	0-47 d		47-72 d		72-110 d		110-141 d		141-163 d	
	2020	2021	2020	2021	2020	2021	2020	2021	2020	2021
N1P1	0.85d	0.85c	32.20e	24.40e	108.95e	102.11e	143.55e	118.71e	60.00e	49.09d
N1P2	1.06c	1.06b	36.40d	30.40d	121.05d	117.11d	159.35d	128.39d	66.82d	54.09c
N2P1	1.06c	1.06b	39.60c	35.60c	131.05c	106.05c	172.90c	158.71bc	72.27c	75.27b
N2P2	1.49a	1.28a	50.00a	46.80a	165.53a	135.79a	218.39a	234.84a	91.36a	85.45a
N3P1	1.28b	1.06b	43.60b	42.80b	145.00b	120.79b	191.29b	163.55b	80.00b	78.18b
N3P2	1.28b	1.06b	39.20c	36.00c	130.79c	124.21b	172.26c	140.00cd	72.27c	56.82c
N	***	***	***	***	***	***	***	***	***	ns
P	***	*	***	***	***	***	***	**	***	*
N*P	***	***	***	***	***	***	***	**	***	ns

DAS, day after seeding. Different letters in each column indicate significant differences between different treatments at same growth stage at P<0.05 level. *significant at P<0.05, **significant at P<0.01, ***significant at P<0.001. ns, no significant and P>0.05.

### Leaf chlorophyll

**Figure 3:**
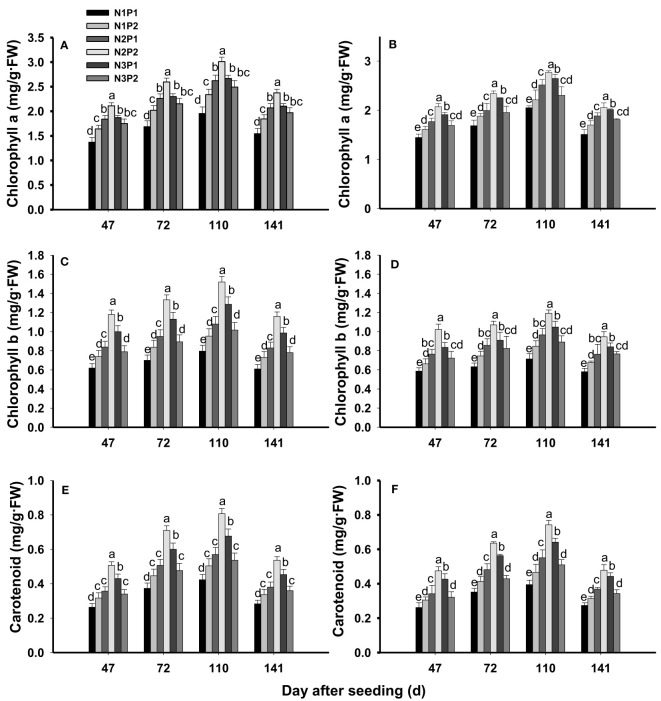
Effects of different nitrogen and phosphorus combinations on contents of chlorophyll a, chlorophyll b and carotenoid of sesbania pea at different growth stages. Different lowercase letters indicate significant differences between different treatments at same growth stage at P< 0.05 level. **(A, C, E)**: 2020; **(B, D, F)**: 2021.

Leaf chlorophyll a, chlorophyll b and carotenoid were significantly affected by N, P fertilizer treatment and the interaction between N and P (*P*=0.0020, **Figure 3**). The contents of chlorophyll a (**Figures 3A, B**), chlorophyll b (**Figures 3C, D**) and carotenoid (**Figures 3E, F**) were significantly increased with increased N and P rates in saline soils (*P*=0.0020). At the level of P1, chlorophyll a, chlorophyll b and carotenoid were significantly improved with increased N rate (*P*=0.0024), and the highest value was showed under N3. At the level of P2, chlorophyll a, chlorophyll b and carotenoid were increased with increased N rate and the maximum value were performed under N2. In addition, the contents of chlorophyll a, chlorophyll b and carotenoid were not significantly different between N1P2 and N3P2 (*P*=0.0080). Consistently, the maximum contents of chlorophyll a, chlorophyll b and carotenoid were all showed under N2P2 (**Figure 3**). Among of the growth stages, the highest contents of chlorophyll a, chlorophyll b and carotenoid were generated at 110 DAS (**Figure 3**).

### POD, CAT and SOD

**Figure 4:**
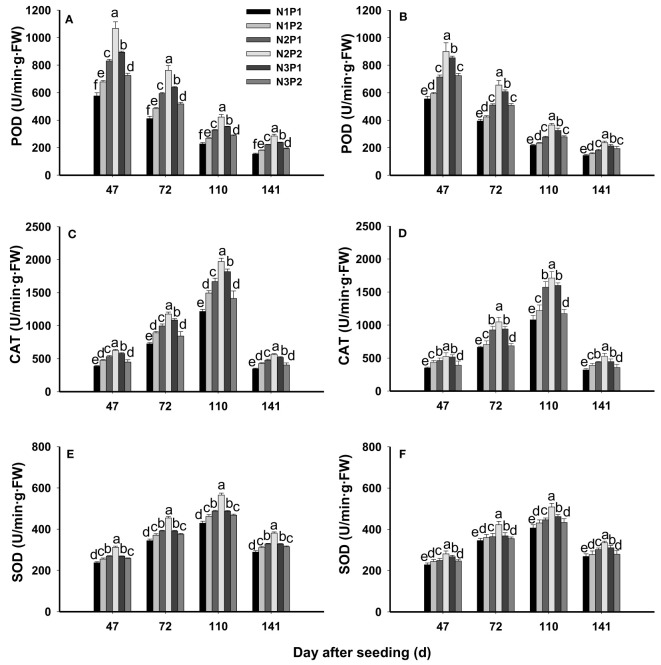
Effects of different nitrogen and phosphorus combinations on activities of peroxidase (POD), catalase (CAT) and superoxide dismutase (SOD) of sesbania pea at different growth stages. Different letters indicate significant differences between different treatments at same growth stage at P<0.05 level. **(A, C, E)**: 2020; **(B, D, F)**: 2021.

The POD activity was significantly affected by N, P fertilizer and their interaction at all growth stages (*P*=0.0003, **Figures 4A, B**). At each growth stage, the POD activity was significantly improved with increased N and P rates (*P*=0.0003), and the highest value was showed under N2P2. Compared with N1P1, POD activity was increased by 12.2%, 36.4%, 73.7%, 54.5% and 28.1% under N1P2, N2P1, N2P2, N3P1 and N3P2 at 47 DAS, respectively. In addition, the POD activity was by 12.7%, 36.9%, 75.9%, 54.6% and 21.5% at 72 DAS, and by 12.6%, 36.0%, 76.7%, 52.5% and 26.9% at 110 DAS compared with N1P1 under each treatment. Furthermore, it was increased by 13.4%, 35.1%, 75.2%, 51.8% and 29.9% at 141DAS, respectively. Among of the growth stages, the highest activity of POD was showed at 47 DAS (**Figures 4A, B**).

The CAT activity was significantly affected by N, P fertilizer treatment and their interaction (*P*=0.0001, **Figures 4C, D**). Similarly, the CAT activity was prominently increased by N and P rate increased. Among the treatments, N2P2 showed the highest CAT activity at each growth stage, which was 56.0%, 26.2%, 15.8%, 5.2% and 37.0% higher than that under N1P1, N1P2, N2P1, N3P1 and N3P2 at 46 DAS, and 60.1%, 39.3%, 15.7%, 9.9% and 45.5% higher at 72 DAS, and 60.5%, 35.7%, 17.0%, 7.8% and 42.5% higher at 110 DAS, and 62.9%, 33.3%, 18.6%, 12.7% and 43.1% higher at 141 DAS than other treatments, respectively. In addition, during the growth periods, the maximum CAT activity was showed at 110 DAS under each treatment (**Figures 4C, D**).

The SOD activity was significantly affected by N, P fertilizer treatment and the interaction between N and P (*P*=0.0001, **Figures 4E, F**). The SOD activities were significantly improved by N and P application rate (*P*=0.0001), and the highest activity was showed under N2P2. Overall, SOD activity gradually increased as the growth period, and the maximum value was produced at 110 DAS. Compared with N1P1, SOD activity was increased by 7.3%, 11.4%, 27.6%, 15.1% and 8.6% under N1P1, N1P2, N2P1, N3P1 and N3P2 at 47 DAS, respectively. Similarly, it was 5.9%, 9.6%, 27.0%, 10.0 and 5.9% higher under N1P1, N1P2, N2P1, N3P1 and N3P2 than N1P1 at 72 DAS. In addition, SOD activity was improved by 6.8%, 11.8%, 28.5%, 13.5% and 7.9% under N1P1, N1P2, N2P1, N3P1 and N3P2 compared with N1P1 at 110 DAS, respectively. Furthermore, it was increased by 5.7%, 13.2%, 28.5%, 14.6% and 6.6% under each treatment compared with N1P1 at 141 DAS, respectively (**Figures 4E, F**).

### Soluble protein and MDA

**Figure 5:**
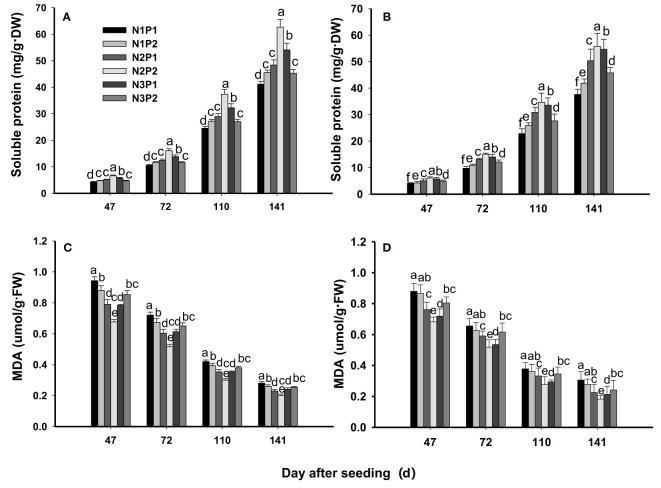
Effects of different nitrogen and phosphorus combinations on contents of soluble protein and malondialdehyde (MDA) of sesbania pea at different growth stages. Different letters indicate significant differences between different treatments at same growth stage at *P*<0.05 level. **(A, C)**: 2020; **(B, D)**: 2021.

The soluble protein content was significantly affected by N, P fertilizer and the interaction between N and P at all growth stages (*P*=0.0053, **Figures 5A, B**). N and P fertilizer significantly improved the content of soluble protein at all the growth stages (*P*=0.0053), and the highest content of soluble protein was showed under the combination treatment of N2P2. Among the treatments, the content of soluble protein was performed N2P2 > N3P1 > N2P1 > N1P2 > N3P2 > N1P1 at all growth stages. Compared with N1P1, the content of soluble protein was increased by 7.1%, 21.4%, 50.0%, 35.7% and 14.3% on 47th DAS, by10.8%, 25.5%, 52.9%, 36.3% and 15.7% on 72nd DAS, by 11.8%, 26.2%, 51.9%, 38.8% and 15.2% on 110th DAS, and by 12.2%, 25.4%, 50.3%, 38.1% and 15.5% on 141st DAS under N1P2, N2P1, N2P2, N3P1 and N3P2, respectively. In addition, the maximum content of soluble protein was showed on 141st DAS under all treatments (**Figures 5A, B**).

The MDA content was significantly affected by N, P fertilizer and by the interaction between N and P fertilizer at all growth stages (*P*=0.0029, **Figures 5C, D**). The MDA content was decreased significantly with increased N and P fertilizer rates (*P*=0.0029). Compared with N1P1, the MDA content decreased by 4.4%, 14.3%, 25.3%, 17.6% and 8.8% on 47th DAS, by 2.9%, 13.0%, 24.6%, 17.4% and 8.7% on 72nd DAS, by 5.0%, 15.0%, 27.5%, 20.0% and 10.0% on 110th DAS, and by 6.9%, 20.7%, 34.5%, 20.7% and 20.7% on 141st DAS under N1P2, N2P1, N2P2, N3P1 and N3P2, respectively. Overall, the lowest content of MDA was showed under N2P2 at each growth stage. In addition, the MDA content was gradually decreased as the growth proceed and the minimum content of MDA was showed on 141st DAS (**Figures 5C, D**).

### Correlation analysis

The correlation analysis showed that the antioxidant enzymes (SOD, POD, CAT) were positively correlated with plant height (*P*=0.0050, R^2^ = 0.910; *P*=0.0009, R^2^ = 0.964; and *P*=0.0150, R^2^ = 0.809), fresh biomass yield (*P*=0.0017, R^2^ = 0.937; *P*=0.0009, R^2^ = 0.954 and *P*=0.0124, R^2^ = 0.831), dry biomass yield (*P*=0.0014, R^2^ = 0.903; *P*=0.0010, R^2^ = 0.952; and *P*=0.0086, R^2^ = 0.855), RGR (*P*=0.0013, R^2^ = 0.954; *P*=0.0024, R^2^ = 0.964; and *P*= 0.0081, R^2^ = 0.808), and soluble protein (*P*=0.0076, R^2^ = 0.873; *P*=0.0012, R^2^ = 0.946; and *P*=0.0030, R^2^ = 0.912), but negatively correlated with MDA (*P*=0.0044, R^2^ = 0.909; *P*=0.0003, R^2^ = 0.978, and *P*=0.0082, R^2^ = 0.858) (**Table 5**). Oppositely, MDA was negatively correlated with plant height (*P*=0.0004, R^2^ = 0.977), fresh biomass yield (*P*=0.0024, R^2^ = 0.922), dry biomass yield (*P*=0.0022, R^2^ = 0.926) and soluble protein (*P*=0.0029, R^2^ = 0.922) (**Table 5**). In addition, there was a positively correlation between soluble protein and plant height (*P*=0.0021, R^2^ = 0.933), fresh biomass yield (*P*=0.0047, R^2^ = 0.904), dry biomass yield (*P*=0.0034, R^2^ = 0.916), and RGR (*P*=0.0052, R^2^ = 0.900) (**Table 5**).

**Table 5:** The correlations (*R^2^*) of antioxidant enzymes with growth and of sesbania pea across growth stages under different nitrogen and phosphorus combinations.

	PH	FW	DW	RGR	SOD	POD	CAT	MDA	SP
**2020**									
**PH**	–								
**FW**	0.947**	–							
**DW**	0.904**	0.998***	–						
**RGR**	0.852**	0.998***	0.998***	–					
**SOD**	0.976***	0.922**	0.836**	0.922**	–				
**POD**	0.933**	0.964***	0.964***	0.964***	0.931**	–			
**CAT**	0.787*	0.878**	0.878**	0.880**	0.830*	0.947**	–		
**MDA**	0.956***	0.916**	0.916**	0.918**	0.962***	0.966***	0.880**	–	
**SP**	0.904**	0.956***	0.956***	0.955***	0.924**	0.956***	0.914**	0.887**	–
**2021**									
**PH**	–								
**FW**	0.922**	–							
**DW**	0.927**	0.996***	–						
**RGR**	0.889**	0.976***	0.990***	–					
**SOD**	0.843**	0.951***	0.970***	0.986***	–				
**POD**	0.994***	0.943**	0.939**	0.964***	0.848**	–			
**CAT**	0.830*	0.785*	0.832*	0.880**	0.870**	0.796*	–		
**MDA**	0.998***	0.927**	0.935**	0.903**	0.856**	0.990***	0.835*	–	
**SP**	0.962***	0.852**	0.876**	0.845**	0.821*	0.935**	0.910**	0.956***	–

PH, plant height; FW, fresh biomass weight; DW, dry biomass weight; RGR, relative growth rate; SOD, superoxide dismutase; POD, peroxidase; CAT, catalase; MDA, malonaldehyde; SP, soluble protein. *significant at P<0.05, **significant at P<0.01, ***significant at P<0.001. The values with italic in table means negatively correlation.

## Discussion

In the present study, plant growth and physiological traits of sesbania pea were significantly affected by N and P fertilizer application in saline soils. N and P are the essential elements required for plant growth, but deficiencies of N and P are common in saline soils. On the contrary, superfluous N and P in soil can inhibit K uptake and aggravate salinity stress, lead to both pH and EC increased (Zhu et al., 2020). Plant responses to salinity stress is a complex network, in which nutrients uptake and photosynthesis are mainly affected under salt stress (Chen et al., 2010; Wang et al., 2011). It was reported that nitrogen promoted seedling growth and yield of crop mainly by improving root activity, photosynthetic productivity, and chlorophyll fluorescence under salt stress (Muhi et al., 2019; Song et al., 2019). In this study, N application generated a higher plant growth, more biomass yield, higher RGR, and chlorophyll content, which might attribute to nitrogen enhanced nutrients uptake and photosynthetic capacity of plant in salinity conditions. In addition, N application could enhance antioxidant enzyme activity and osmoregulation substances accumulation under saline conditions (Song et al., 2019). That was why the antioxidative enzymes (SOD, POD, CAT) and osmotic substances (soluble protein) were significantly increased in the fertilizer treatments in the present study. Similar results were also observed in our previous studies (Zhu et al., 2020)and by Song et al. (2019). The increased SOD, POD and CAT could alleviate the damage of ROS (Reactive Oxygen Species) that generate under salt stress on DNA, proteins, pigments and membranes(Jayakumar et al., 2014). In addition, it has been confirmed that the osmotic substances could enhance water uptake by increasing positive osmotic potential in soil, which could trigger chemical signaling that leads to increased stomatal aperture and photosynthetic rate (Zhou et al., 2014). In the present study, the correlation analysis showed that the antioxidant enzymes (SOD, POD, CAT) were positively correlated with biomass yield (fresh biomass yield and dry biomass yield) (**Figures S1, S3, S5, S7**), but MDA was negatively correlated with plant growth (plant height), biomass yield and antioxidant enzymes (**Figures S2, S4, S6, S8**), and antioxidant enzymes negatively correlated with MDA at each growth stage and across the growth period (**Table 5**). Similar results were found in the study of Ali et al. (2019) and Zhu et al. (2020) in other crops. These results confirmed our hypothesis that productivity of sesbania pea in saline soils could be improved by enhancing antioxidant capacity with optimum application of nitrogen and phosphate combination. Furthermore, there was no strong differences for most of the results in the two years of experimentation, the possible reason was that the environment factors was very similar between the two years as showed in **Figure 1**, the average daily temperature during the growing season (from May to November) was 21.9°C and 22.0°C, the daily rainfall was 4.20 mm and 4.54 mm, the mean daily sunshine hour was 4.50 and 4.93 h day^−1^ in 2020 and 2021, respectively. On the other hand, the two year’s experiments were conducted at the same field.

In saline conditions, P is usually more deficient than N(Delgado-Baquerizo et al., 2013). In saline soils, plants usually suffer from P deficiency because of low availability, as showed in **Table 1**, which resulting in inhibited growth. In other words, salt stress of plant could be alleviated by P application. That was the reason that plant growth, biomass yield and physiological traits were significantly improved when P rate increased from P1 to P2 at same N rate in the present study. Manimaran and Poonkodi (2014) also found that phosphorus alleviated salt stress in maize in salinity conditions. Under salinity conditions, P application could increase absorption of phosphorus, potassium and other elements in the root, which can delay leaf senescence and enhance nutrient uptake, resulting in a fast growth and biomass production as showed in this study (Li et al., 2013; Wang et al., 2017). In the present study, growth and physiological trait was prominently improved with N rate increased at each P level. This indicated that N may played a complementary role in the uptake of P in saline soils. This result supported by our previous study that the growth and yield of castor (*Riciuns communis* L.) was significantly higher under N-P combination application than N or P sole application in saline soils(Wu et al., 2019). In addition, only two P rates were tested in this study, and the values of parameters was higher under P2 than that under P1 at each N level. Whether P2 (180 kg·hm^-2^) is the optimum rate for sesbania pea in saline soils or not should be further studied.

However, N application may lead to excessive or inadequate nutrient uptake or even give rise to some abiotic stresses such as drought and secondary salinity(Zhu et al., 2020). As shown in this study, most of the parameters were improved from N1 rate to N2 rate but decreased from N2 rate to N3 rate at each P level. In this study, N2 rate (360 kg·hm^-2^) was appropriate for sesbania pea in saline soils. Excessive fertilizer input leads to low use efficiency due to rapid fertilizer loss, such as N losses through ammonia volatilization, denitrification, surface running-off, and leaching in the soil-flood water system(DeDatta and Buresh, 1998). A low nutrition use efficiency also results in environmental pollution, such as soil acidification (Guo et al., 2010), air pollution (Smil, 1999), and water eutrophication (Diaz and Rosenberg, 2008). Furthermore, excessive N application can increase soil electronic conductivity and aggravate salinity stress (Zhu et al., 2020). Appropriate fertilizer application can promote both plant growth and yield and alleviate salt stress(Peng et al., 2002). In the present study, most of the growth and physiological traits showed the maximum values in the N2P2 treatment at each growth stage. This suggested that the optimum fertilizer combination was 360 kg·hm^-2^ N combined with 180 kg·hm^-2^ P for sesbania pea in saline soils.

## Conclusion

The results of present study revealed that N and P application significantly improved plant growth and associated physiological traits of sesbania pea in saline soils. The maximum values of plant growth and biomass yield were consistently produced under N2P2 treatment (360 kg·hm^-2^ N + 180 kg·hm^-2^ P) at each growth stage. In light of the present findings, it can be concluded that the productivity of sesbania pea was improved in saline soils by enhancing antioxidant capacity with optimum application of nitrogen and phosphate combination of 360 kg·hm^-2^ N and 180 kg·hm^-2^ P for sesbania pea in saline soils. Theoretically, the salinity declined to a certain degree (EC declined from 10.87 to 8.35 mS cm^-1^) after two years of cultivating sesbania pea and the combined fertilization of nitrogen and phosphate fertilizers.

## Data availability statement

The original contributions presented in the study are included in the article/**Supplementary Material**. Further inquiries can be directed to the corresponding author.

## Author contributions

Data curation, GLZ and YX. Formal analysis, YX. Investigation, GLZ, YX and ZX. Methodology, YX and GSZ. Resources, GSZ and NN. Software, ZX and IA. Supervision, GSZ and NN. Validation, IA. Writing – original draft, GLZ. Writing – review and editing, NN and GSZ. All authors contributed to the article and approved the submitted version.

## Funding

This work was partially funded by National Key Research and Development Program of China (2022YFE0113400, 2018YFE0108100), Special Found Project for Technology on Carbon Peak Carbon-Neutral in 2022, Jiangsu Province (BE2022305), Key Laboratory of Digital Upland Crops of Zhejiang Province (2022E10012), the Natural Science Foundation of Jiangsu Province of China (BK20221371), Postgraduate Research & Practice Innovation Program of Jiangsu Province (SJCX22_1784, SJCX21_1624), the Open Project Program of Joint International Research Laboratory of Agriculture and Agri-Product Safety, the Ministry of Education of China, Yangzhou University (JI-LAR-KF202004, JILAR-KF202106), the Innovation and Promotion of Forestry Science and Technology Program of Jiangsu Province (LYKJ[2019]47).

## Conflict of interest

The authors declare that the research was conducted in the absence of any commercial or financial relationships that could be construed as a potential conflict of interest.

## Publisher’s note

All claims expressed in this article are solely those of the authors and do not necessarily represent those of their affiliated organizations, or those of the publisher, the editors and the reviewers. Any product that may be evaluated in this article, or claim that may be made by its manufacturer, is not guaranteed or endorsed by the publisher.
