# Supplementary material for: Improving productivity of Sesbania pea in saline soils by enhancing antioxidant capacity with optimum application of nitrogen and phosphate combination
Source: Front Plant Sci. 2022 Nov 2;13:1027227. doi: 10.3389/fpls.2022.1027227 (PMC9667019; doi:10.3389/fpls.2022.1027227)
Supplement: Supplementary file 1 [file DataSheet_1.docx]

**FIGURE S1 |** The correlation between biomass yield (fresh weight and dry weight) and SOD (A, B), POD (C, D) and CAT (E, F) at 47 DAS (seedling stage) in 2020 and 2021, respectively.

*significant at *P*<0.05, ** significant at *P*<0.01, *** significant at *P*<0.001.

**FIGURE S2 |** The correlation between MDA and plant height (A), fresh weight (B), dry weight (C), SOD (D), POD (E) and CAT (F) at 47 DAS (seedling stage) in 2020 and 2021.

*significant at *P*<0.05, ** significant at *P*<0.01, *** significant at *P*<0.001.

**FIGURE S3 |** The correlation between biomass yield (fresh weight and dry weight) and SOD (A, B), POD (C, D) and CAT (E, F) at 72 DAS (squaring stage) in 2020 and 2021, respectively.

*significant at *P*<0.05, ** significant at *P*<0.01, *** significant at *P*<0.001.

**FIGURE S4 |** The correlation between MDA and plant height (A), fresh weight (B), dry weight (C), SOD (D), POD (E) and CAT (F) at 72 DAS (squaring stage) in 2020 and 2021.

*significant at *P*<0.05, ** significant at *P*<0.01, *** significant at *P*<0.001.

**FIGURE S5 |** The correlation between biomass yield (fresh weight and dry weight) and SOD (A, B), POD (C, D) and CAT (E, F) at 110 DAS (flowering stage) in 2020 and 2021, respectively.

*significant at *P*<0.05, ** significant at *P*<0.01, *** significant at *P*<0.001.

**FIGURE S6 |** The correlation between MDA and plant height (A), fresh weight (B), dry weight (C), SOD (D), POD (E) and CAT (F) at 110 DAS (flowering stage) in 2020 and 2021.

*significant at *P*<0.05, ** significant at *P*<0.01, *** significant at *P*<0.001.

**FIGURE S7 |** The correlation between biomass yield (fresh weight and dry weight) and SOD (A, B), POD (C, D) and CAT (E, F) at 141 DAS (pod bearing stage) in 2020 and 2021, respectively.

*significant at *P*<0.05, ** significant at *P*<0.01, *** significant at *P*<0.001.

**FIGURE S8 |** The correlation between MDA and plant height (A), fresh weight (B), dry weight (C), SOD (D), POD (E) and CAT (F) at 141 DAS (pod bearing stage) in 2020 and 2021.

*significant at *P*<0.05, ** significant at *P*<0.01, *** significant at *P*<0.001.
